# Supplementary material for: Non-equilibrium active noise enhances generative memory in diffusion models
Source: Res Sq. 2026 Feb 5:rs.3.rs-8702780. Preprint. [Version 1] doi: 10.21203/rs.3.rs-8702780/v1 (PMC12889839; doi:10.21203/rs.3.rs-8702780/v1)
Supplement: 1 [file NIHPPRS8702780V1-supplement-1.pdf]

## A1. DERIVATION FOR ACTIVE REVERSE DIFFUSION

The forward active diffusion process for $\mathbf{x}(t), \boldsymbol{\eta}(t) \in \mathbb{R}^d$ is defined, for $t \in [0, t_f]$, by the system of stochastic differential equations (SDEs)

$$\dot{\mathbf{x}} = -k\mathbf{x} + \boldsymbol{\eta}(t) + \boldsymbol{\xi}_1(t) \ , \tag{A1}$$

$$\dot{\boldsymbol{\eta}} = -\frac{\boldsymbol{\eta}}{\tau} + \boldsymbol{\xi}_2(t) \ , \tag{A2}$$

where $\boldsymbol{\xi}_1, \boldsymbol{\xi}_2$ are independent Gaussian white noise with, for all $i, j \in \{1, \ldots, d\}$,

$$\langle \xi_{1,i}(t) \rangle = 0 \ , \quad \langle \xi_{2,i}(t) \rangle = 0 \ , \tag{A3}$$

$$\langle \xi_{1,i}(t)\xi_{1,j}(t') \rangle = 2T_p \delta_{ij}\delta(t - t') \ , \tag{A4}$$

$$\langle \xi_{2,i}(t)\xi_{2,j}(t') \rangle = \frac{2T_a}{\tau^2}\delta_{ij}\delta(t - t') \ . \tag{A5}$$

Here the angle bracket $\langle \cdots \rangle$ denotes the ensemble average over the noise realizations $(\boldsymbol{\xi}_1, \boldsymbol{\xi}_2)$. Unless otherwise stated, we assume $k\tau \neq 1$ throughout the paper.

The initial condition for the forward process is the joint distribution between the data degrees of freedom $(\mathbf{x})$ and the corresponding active degrees of freedom $(\boldsymbol{\eta})$, and it is constructed as

$$P_0(\mathbf{x}_0, \boldsymbol{\eta}_0) = P_0(\mathbf{x}_0)P_0(\boldsymbol{\eta}_0) \tag{A6}$$

where we assume

$$P_0(\boldsymbol{\eta}_0) = \mathcal{N}(\boldsymbol{\eta}_0; \mathbf{0}, \tau^{-1}T_a I_d) \propto \exp\left(-\frac{\tau}{2T_a}\|\boldsymbol{\eta}_0\|^2\right) \tag{A7}$$

and $P_0(\mathbf{x}_0)$ is the distribution from which the data is drawn. Here $\|\cdot\|$ denotes the standard Euclidean norm. With these initial conditions, the conditional distribution $P(\mathbf{x}, \boldsymbol{\eta}|\mathbf{x}_0, \boldsymbol{\eta}_0; t)$ is given by (see, Sec. A2 2)

$$P(\mathbf{x}, \boldsymbol{\eta}|\mathbf{x}_0, \boldsymbol{\eta}_0; t) = \mathcal{N}(\vec{X}_t; \vec{\mu}_t, C_t) \propto \exp\left[-\frac{(\vec{X}_t - \vec{\mu}_t)^\top C_t^{-1}(\vec{X}_t - \vec{\mu}_t)}{2}\right] \tag{A8}$$

where we have defined the state vector $\vec{X}_t = \begin{pmatrix} \mathbf{x}_t \\ \boldsymbol{\eta}_t \end{pmatrix} \in \mathbb{R}^{2d}$ and

$$\vec{\mu}_t = \begin{pmatrix} e^{-kt}\mathbf{x}_0 + \frac{e^{-t/\tau} - e^{-kt}}{k - \frac{1}{\tau}}\boldsymbol{\eta}_0 \\ e^{-t/\tau}\boldsymbol{\eta}_0 \end{pmatrix} \tag{A9}$$

$$C_t = \begin{pmatrix} m_{11} & m_{12} \\ m_{12} & m_{22} \end{pmatrix} \otimes I_d \tag{A10}$$

$$m_{11} = \frac{T_p}{k}(1 - a^2) + \frac{T_a}{\tau^2}\left(\frac{\tau}{kc} + \frac{1}{d^2}\left(\frac{4ab}{c} - b^2\tau - \frac{a^2}{k}\right)\right) \tag{A11}$$

$$m_{12} = \frac{T_a}{\tau c d}\left(k(1 - b^2) - \frac{1}{\tau}\left(1 + b^2 - 2ab\right)\right) \tag{A12}$$

$$m_{22} = \frac{T_a}{\tau}(1 - b^2) \tag{A13}$$

$$a = e^{-kt}, \quad b = e^{-t/\tau}, \quad c = k + \frac{1}{\tau}, \quad d = k - \frac{1}{\tau} \ , \tag{A14}$$

where $\cdot^\top$ denotes the transpose operation, $\otimes$ the Kronecker product, and $I_d$ the identity in $\mathbb{R}^{d \times d}$. Marginalizing the conditional probability on the initial distribution $P_0(\mathbf{x}_0, \boldsymbol{\eta}_0) = P_0(\boldsymbol{x}_0)P_0(\boldsymbol{\eta}_0)$ gives the unconditioned distribution at time $t$:

$$P(\mathbf{x}, \boldsymbol{\eta}; t) = \int P(\mathbf{x}, \boldsymbol{\eta}|\mathbf{x}_0, \boldsymbol{\eta}_0; t)P_0(\mathbf{x}_0, \boldsymbol{\eta}_0)\, d\mathbf{x}_0 d\boldsymbol{\eta}_0 \ . \tag{A15}$$

One admissible reverse diffusion SDE for this process is given by ([9, 19])

$$-\dot{\mathbf{x}} = -k\mathbf{x} + \boldsymbol{\eta} + 2T_p \mathscr{F}_{\mathbf{x}}(\mathbf{x}, \boldsymbol{\eta}; t) + \boldsymbol{\xi}_1(t) \tag{A16}$$

$$-\dot{\boldsymbol{\eta}} = -\frac{\boldsymbol{\eta}}{\tau} + \frac{2T_a}{\tau^2}\mathscr{F}_{\boldsymbol{\eta}}(\mathbf{x}, \boldsymbol{\eta}; t) + \boldsymbol{\xi}_2(t) \tag{A17}$$

where $\mathscr{F}_{\mathbf{x}}(\mathbf{x}, \boldsymbol{\eta}; t) \equiv \nabla_{\mathbf{x}} \log P(\mathbf{x}, \boldsymbol{\eta}; t)$ and $\mathscr{F}_{\boldsymbol{\eta}}(\mathbf{x}, \boldsymbol{\eta}; t) \equiv \nabla_{\boldsymbol{\eta}} \log P(\mathbf{x}, \boldsymbol{\eta}; t)$ are the score functions for this process.

Using Eq. (A15), we have

$$\begin{aligned}\begin{pmatrix}\mathscr{F}_{\boldsymbol{x}}(\mathbf{x}, \boldsymbol{\eta}; t)\\ \mathscr{F}_{\boldsymbol{\eta}}(\mathbf{x}, \boldsymbol{\eta}; t)\end{pmatrix} &= \frac{1}{P(\mathbf{x}, \boldsymbol{\eta}; t)} \int \big[-C_t^{-1}\vec{X}\big] P(\mathbf{x}, \boldsymbol{\eta}|\mathbf{x}_0, \boldsymbol{\eta}_0; t) P_0(\mathbf{x}_0, \boldsymbol{\eta}_0)\, d\mathbf{x}_0 d\boldsymbol{\eta}_0 \\ &= \int \big[-C_t^{-1}\vec{X}\big] P(\mathbf{x}_0, \boldsymbol{\eta}_0|\mathbf{x}, \boldsymbol{\eta}; t)\, d\mathbf{x}_0 d\boldsymbol{\eta}_0 \ ,\end{aligned} \tag{A18}$$

where we have defined the conditional posterior distribution $P(\mathbf{x}_0, \boldsymbol{\eta}_0|\mathbf{x}, \boldsymbol{\eta}; t) = P(\mathbf{x}, \boldsymbol{\eta}|\mathbf{x}_0, \boldsymbol{\eta}_0; t) P_0(\mathbf{x}_0, \boldsymbol{\eta}_0)/P(\mathbf{x}, \boldsymbol{\eta}; t)$ based on the Bayes' rule. We then obtain the expressions for the loss functions corresponding to the scores:

$$\mathscr{F}_{\mathbf{x}}(\mathbf{x}, \boldsymbol{\eta}; t) = \frac{-m_{22}(\mathbf{x} - a\langle\mathbf{x}_0\rangle_t - \frac{a-b}{d}\langle\boldsymbol{\eta}_0\rangle_t) + m_{12}(\boldsymbol{\eta} - b\langle\boldsymbol{\eta}_0\rangle_t)}{\Delta_t} \tag{A19}$$

$$\mathscr{F}_{\boldsymbol{\eta}}(\mathbf{x}, \boldsymbol{\eta}; t) = \frac{-m_{11}(\boldsymbol{\eta} - b\langle\boldsymbol{\eta}_0\rangle_t) + m_{12}(\mathbf{x} - a\langle\mathbf{x}_0\rangle_t - \frac{a-b}{d}\langle\boldsymbol{\eta}_0\rangle_t)}{\Delta_t} \tag{A20}$$

where $\Delta_t \equiv \det\begin{pmatrix} m_{11} & m_{12}\\ m_{12} & m_{22}\end{pmatrix} = m_{11}m_{22} - m_{12}^2$ and $\langle\cdots\rangle_t$ denotes the expectation over the backward conditional distribution $\mathbb{E}_{(\mathbf{x}_0, \boldsymbol{\eta}_0)\sim P(\cdot|\mathbf{x}, \boldsymbol{\eta}; t)}[\cdots] = \int d\mathbf{x}_0 d\boldsymbol{\eta}_0 \cdots P(\mathbf{x}_0, \boldsymbol{\eta}_0|\mathbf{x}, \boldsymbol{\eta}; t)$.

Setting $T_p = 0$ in the active process allows us to ignore $\mathscr{F}_{\mathbf{x}}$ and learn only $\mathscr{F}_{\boldsymbol{\eta}}$. See Ref. [25] for a discussion on why $\mathscr{F}_{\mathbf{x}}$ is more difficult to learn.

## A2. NUMERICAL IMPLEMENTATION

### 1. Extension of the CLD [25] Code

Here we discuss the numerical implementation of reverse diffusion process using a score objective and the architectures of the neural networks used. We adapt the implementation from Ref. [25]. First, the initial data, $(\mathbf{x}_0, \boldsymbol{\eta}_0) \sim P(\mathbf{x}_0, \boldsymbol{\eta}_0)$, are perturbed in the forward process over a time interval, $t \sim \mathscr{U}[0, t_f]$, where $\mathscr{U}$ is the uniform distribution and $t_f$ is the length of time for which the forward process is run. The perturbation kernel for active diffusion is given in Sec. A2 2. For $t_f \to \infty$, the data distribution reduces to a multidimensional Gaussian distribution centered at the origin. Using these generated samples at different times, we train the neural network to minimize the score objective, a hybrid score matching objective in our case [37]. To "inform" the neural network about the structure of the score, we use a mixed score parameterization [25, 37] (Sec. A2 3).

The primary difference between CLD and active diffusion is the stochastic differential equations (SDEs) defining each process. Thus, implementing active diffusion was achieved by sub-classing the existing CLD class and overriding key methods such as the SDEs of the diffusion process and the perturbation kernel functions.

### 2. Perturbation Kernel for Active Diffusion

Denoting the state vector by $\vec{X}_t = (\mathbf{x}_t^\top, \boldsymbol{\eta}_t^\top)^\top \in \mathbb{R}^{2d}$, the forward active diffusion process (A1)–(A5) can be recast in the following form:

$$d\vec{X}_t = M\vec{X}_t dt + G d\vec{w}_t \tag{A21}$$

$$M = \begin{pmatrix}-k & 1\\ 0 & -1/\tau\end{pmatrix} \otimes I_d \ ,\ G = \begin{pmatrix}\sqrt{2T_p} & 0\\ 0 & \sqrt{2T_a}/\tau\end{pmatrix} \otimes I_d \ , \tag{A22}$$

where $\vec{w}_t$ is a standard Wiener process in $\mathbb{R}^{2d}$. The solution to this equation can be formally written as

$$\vec{X}_t = e^{Mt}\vec{X}_0 + \int_0^t e^{M(t-s)} G\, d\vec{w}_s \ . \tag{A23}$$

Denoting the expectations with respect to the Wiener noise by $\langle \cdots \rangle$, we have $\langle \vec{X}_t \rangle = e^{Mt}\vec{X}_0$ and

$$\langle \vec{X}_t \vec{X}_t^\top \rangle = e^{Mt}\vec{X}_0\vec{X}_0^\top e^{M^\top t} + \int_0^t e^{Ms} GG^\top e^{M^\top s}\, ds\ . \tag{A24}$$

Since in the SDE (A21) the drift is linear and the noise is additive Gaussian, if the data is normally distributed at $t = 0$, it is normally distributed throughout the entire forward process ($t \in [0, t_f]$). Expanding the expressions above, the mean ($\vec{\mu}_t = \langle \vec{X}_t \rangle$) and covariance ($C_t = \langle (\vec{X}_t - \vec{\mu}_t)(\vec{X}_t - \vec{\mu}_t)^\top \rangle = \langle \vec{X}_t \vec{X}_t^\top \rangle - \vec{\mu}_t \vec{\mu}_t^\top$) of the data at various time instants in the forward process are then given by Eqs. (A9)–(A14).

We now set $T_p = 0$. Marginalizing the conditional distribution $P(\mathbf{x}, \boldsymbol{\eta} | \mathbf{x}_0, \boldsymbol{\eta}_0; t)$ (A8) over the initial distribution of active degrees of freedom $P_0(\boldsymbol{\eta}_0)$ (A7), we obtain the partially-conditional distribution

$$P(\vec{X}_t | \mathbf{x}_0; t) = \mathcal{N}(\vec{X}_t; \bar{\vec{\mu}}_t, \bar{C}_t) \propto \exp\left[ -\frac{(\vec{X}_t - \bar{\vec{\mu}}_t)^\top \bar{C}_t^{-1} (\vec{X}_t - \bar{\vec{\mu}}_t)}{2} \right] \tag{A25}$$

where the mean vector and the covariance matrix are given respectively by

$$\bar{\vec{\mu}}_t = \begin{pmatrix} e^{-kt}\mathbf{x}_0 \\ \mathbf{0} \end{pmatrix} \tag{A26}$$

$$\bar{C}_t = \begin{pmatrix} \bar{m}_{11} & \bar{m}_{12} \\ \bar{m}_{12} & \bar{m}_{22} \end{pmatrix} \otimes I_d \tag{A27}$$

with

$$\bar{m}_{11} = \frac{T_a}{\tau}\left( \frac{1 - a^2}{kc} - \frac{2}{cd}(ab - a^2) \right) = \frac{T_a}{\tau}\left( \frac{1}{kc} + \frac{a^2}{kd} - \frac{2}{cd}ab \right) \tag{A28}$$

$$\bar{m}_{12} = \frac{T_a}{\tau c}(1 - ab) \tag{A29}$$

$$\bar{m}_{22} = \frac{T_a}{\tau} \tag{A30}$$

$$a = e^{-kt},\ b = e^{-t/\tau},\ c = k + \frac{1}{\tau},\ d = k - \frac{1}{\tau}\ . \tag{A31}$$

Here $\bar{\cdot}$ represents the partially-marginalized quantities.

The reason for this partial marginalization will become clear in the next section where we use hybrid score matching (HSM) for the active process [25, 37].

### 3. Hybrid Score Matching and Mixed Score Parametrization

For the 2D Swiss Roll, multiple Gaussians and 2D alanine dipeptide datasets, we use the hybrid score matching objective for training the neural network. For the 25D alanine dipeptide and 2D Ising model datasets, we use the score-mixing objective for training (see Sec. A2).

The score matching objective that the neural network needs to optimize is given in Eq. A20,

$$\mathcal{L}(w) = \mathbb{E}_{t \sim \mathcal{U}[0,t_f], \vec{X}(t) \sim P(\vec{X}(t))} \left[ ||S_{\mathbf{w}}^{(\boldsymbol{\eta})}(\vec{X}) - \mathscr{F}_{\boldsymbol{\eta}}(\vec{X}(t))||^2 \right] \tag{A32}$$

$$= \mathbb{E}_{t \sim \mathcal{U}[0,t_f], \vec{X}(t) \sim P(\vec{X}(t))} \left[ ||S_{\mathbf{w}}^{(\boldsymbol{\eta})}(\vec{X}) - \nabla_{\boldsymbol{\eta}} P(\vec{X}(t))||^2 \right] . \tag{A33}$$

Ref. [37] showed that for a forward process conditioned on additional degrees of freedom apart from the data, this loss can be replaced with an equivalent function which makes use of the distribution of the additional variables (active degrees of freedom in our case). The new score function is the HSM function, given by

$$\mathcal{L}_{\text{HSM}}(w) = \mathbb{E}_{t \sim \mathcal{U}[0,t_f], \mathbf{x}_0 \sim P(\mathbf{x}_0), \vec{X} \sim P(\vec{X}(t)|\mathbf{x}_0)} \left[ ||S_{\mathbf{w}}^{(\boldsymbol{\eta})}(\vec{X}) - \nabla_{\boldsymbol{\eta}} P(\vec{X}(t)|\mathbf{x}_0)||^2 \right] \tag{A34}$$

We parameterize this score objective (Eq. (A34)), further following Ref. [25], as

$$S_w^{(\boldsymbol{\eta})}(\vec{X}) = -\frac{\boldsymbol{\eta}}{m_{22}} + S_{\mathbf{w}}^{\text{new}(\boldsymbol{\eta})}(\vec{X}) \tag{A35}$$

where $m_{22}$ is the element of the covariance matrix representing the variance in the active degrees of freedom, $\boldsymbol{\eta}$. $S_{\mathbf{w}}^{\text{new}(\boldsymbol{\eta})}(\vec{X})$ is the new parameterization of score objective that the neural network has to learn. This parameterization ensures that the neural network tries to learn only the reverse process for the active degrees since the reverse process for the data degrees is a deterministic process entirely determined by the active degrees.

### 4. Description of Datasets

The 2D alanine dipeptide toy model was implemented by loading in a file with training data and randomly drawing from the imported dataset. For diffusion on the Ising model, each training sample, a 32-by-32 pixel image with 1 channel, was generated by performing 1,000,000 MCMC sampling steps from a random initial configuration. The training data was generated to have discrete values ($-1$ or $+1$), the perturbation kernel was allowed to vary lattice sites in a continuous manner, and the final samples were discretized back to values of $(-1, +1)$ using a cutoff of 0.

### 5. Neural Network Architectures and Model Training Details

In the toy model examples (Gaussian mixtures, Swiss rolls, and 2D alanine dipeptide), the score was learned by a multi-layer perceptron with 4 hidden layers of 128 nodes each. The 25D alanine dipeptide datasets and Ising lattices were treated as images with one channel, and the score was learned using a neural network with the NCSN++ architecture [19].

For the toy models, the batch size for each iteration was 512 training samples; for Ising lattices, the batch size was 1 sample per iteration. After training, all models were used to synthesize 10,000 samples.

### 6. Sampling

All toy models (excepting the 2D alanine dipeptide model) used the Euler-Maruyama sampling scheme. The 2D and 25D alanine dipeptide and Ising models used an ODE sampler with adaptive step size.

In the 2D toy model examples, we disable denoising at the last step of the sampling scheme. Previous diffusion studies observed that a denoising step (in which only the drift term of the reverse SDE was applied, and not the diffusion term) improved the FID scores of generated image samples by removing noise that is otherwise undetectable by the human eye [26]. This positive effect of the last denoising step is most evident in passive diffusion and does not affect the quality of the samples generated by CLD (see Ref. [25]) or, by extension, active diffusion. We disabled denoising in toy models to be able to directly compare the performance of analytic and numeric score functions (Fig. 2), but retain denoising in the 2D alanine dipeptide and Ising model diffusion since these datasets are image-like in nature.

Although we make direct comparisons of the performance of sample generation of active diffusion and CLD using the EM and ODE samplers, we note that CLD was found to perform best using a custom sampling scheme created by the authors of the method [25]. The comparison of performance of active diffusion and CLD using this sampler is intended in future iterations of this work.

## A3. GAUSSIAN MIXTURES AND SWISS ROLLS

### 1. Reverse Diffusion with Analytic Score on Mixtures of Gaussians

The passive forward diffusion process leads to the evolution of the probability density in the following way,

$$P_t(\mathbf{x}) \propto \sum_\alpha \frac{p_\alpha}{\Pi_i \sqrt{h_i^\alpha}} \int Dx_{0,i} \exp\left(-\sum_i \left(\frac{(x_{0,i}-\mu_i^\alpha)^2}{2h_i^\alpha} + \frac{(x_i - a x_{0,i})^2}{2\Delta}\right)\right) \tag{A36}$$

$$= \sqrt{\Delta} \sum_\alpha \frac{p_\alpha}{\Pi_i \sqrt{\Delta + h_i^\alpha a^2}} \exp\left(-\sum_i \frac{(x_i - a\mu_i^\alpha)^2}{2(\Delta + a^2 h_i^\alpha)}\right) \tag{A37}$$

where $a = e^{-kt}$, the index $i$ runs over the different peaks, and the index $\alpha$ runs over the dimensions of the dataset, and $\Delta$ is given as, $\Delta = \frac{T}{k}(1 - e^{-2kt})$. Thus the score function for the reverse process is given by

$$\frac{\partial \log(P_t(\mathbf{x}))}{\partial x_i} = -\frac{1}{P_t(\mathbf{x})} \sum_\alpha \frac{p_\alpha}{\Pi_j \sqrt{\Delta + h_j^\alpha a^2}} \frac{(x_i - a\mu_i^\alpha)}{\Delta + a^2 h_i^\alpha} \exp\left(-\sum_j \frac{(x_j - a\mu_j^\alpha)^2}{2(\Delta + a^2 h_j^\alpha)}\right) \tag{A38}$$

Following the same procedure for the active process yields

$$P_0(\mathbf{x}_0) \propto \sum_\alpha p_\alpha \prod_i \left[ \frac{1}{\sqrt{h_i^\alpha}} \exp\left( -\frac{(x_{0,i} - \mu_i^\alpha)^2}{2h_i^\alpha} \right) \right] \ , \ P_0(\boldsymbol{\eta}_0) \propto \exp\left( -\frac{\boldsymbol{\eta}_0^2}{2g} \right) \tag{A39}$$

$$P(\boldsymbol{x}, \boldsymbol{\eta} | \boldsymbol{x}_0, \boldsymbol{\eta}_0; t) \propto \exp\left( -\frac{\vec{X}^T C^{-1} \vec{X}}{2} \right) \ , \ \vec{X} = \begin{pmatrix} \boldsymbol{x} - a\boldsymbol{x}_0 - b\boldsymbol{\eta}_0 \\ \boldsymbol{\eta} - c\boldsymbol{\eta}_0 \end{pmatrix} \ , \ C = \begin{pmatrix} m_{11} & m_{12} \\ m_{12} & m_{22} \end{pmatrix} \tag{A40}$$

$$P_t(\boldsymbol{x}, \boldsymbol{\eta}) \propto \sqrt{\Delta} g^{dims} \sum_\alpha p_\alpha \prod_i \left( \frac{h_i^\alpha}{\sqrt{\Delta_{\text{eff,i}}^\alpha}} \right) \prod_i \left( \exp\left( -\frac{k_1(x_i - a\mu_i^\alpha)^2 - 2k_2(x_i - a\mu_i^\alpha)\eta_i + k_{3,i}^\alpha \eta_i^2}{2\Delta_{\text{eff,i}}^\alpha} \right) \right) \tag{A41}$$

$$k_1 = c^2 g + m_{22} \ , \ k_2 = bcg + m_{12} \ , \ k_{3,i}^\alpha = b^2 g + a^2 h_i^\alpha + m_{11} \ , \tag{A42}$$

$$\Delta_{\text{eff,i}} = k_1 k_{3,i}^\alpha - k_2^2 \ , \ \Delta = m_{11} m_{22} - m_{12}^2 \tag{A43}$$

$$\frac{\partial \ln(P(\boldsymbol{x}, \boldsymbol{\eta}))}{\partial x_i} = -\frac{1}{P_t(\boldsymbol{x}, \boldsymbol{\eta})} \sum_\alpha \prod_j \left( \frac{h_j^\alpha}{\sqrt{\Delta_{\text{eff,j}}^\alpha}} \right) p_\alpha \frac{k_1(x_i - a\mu_i^\alpha) - k_2 \eta_i}{\Delta_{\text{eff,i}}^\alpha} z_\alpha \tag{A44}$$

$$\frac{\partial \ln(P(\boldsymbol{x}, \boldsymbol{\eta}))}{\partial \eta_i} = -\frac{1}{P_t(\boldsymbol{x}, \boldsymbol{\eta})} \sum_\alpha \prod_j \left( \frac{h_j^\gamma}{\sqrt{\Delta_{\text{eff,j}}^\gamma}} \right) p_\alpha \frac{k_{3,i}^\alpha \eta_i - k_2 x_i}{\Delta_{\text{eff,i}}^\alpha} z_\alpha \tag{A45}$$

$$z_\alpha = \exp\left( -\sum_r \left( \frac{k_1(x_{r - a\mu_r^\alpha})^2 - 2k_2(x_r - a\mu_r^\alpha)\eta_r + k_{3,r}^\alpha \eta_r^2}{2\Delta_{\text{eff,r}}^\alpha} \right) \right) . \tag{A46}$$

Fig. A1 illustrates the effect of reverse diffusion step size on sample synthesis quality when the analytic score function is used. Two datasets are presented, both consisting of 9 Gaussian peaks with standard deviation $\sigma = 0.04$ and differing in the spacing between the peaks. The positions of the means of the Gaussian peaks $(\mu_x^i, \mu_y^i)$ is given by,

$$[(\mu_x, \mu_y)] = \left[ (0,0), (r,0), \left( \frac{r}{\sqrt{2}}, \frac{r}{\sqrt{2}} \right), (0,r), \left( -\frac{r}{\sqrt{2}}, \frac{r}{\sqrt{2}} \right), (-r,0), \left( -\frac{r}{\sqrt{2}}, -\frac{r}{\sqrt{2}} \right), (0,-r), \left( \frac{r}{\sqrt{2}}, -\frac{r}{\sqrt{2}} \right) \right] \tag{A47}$$

where the value of $r$ sets the distance between individual Gaussian peaks. In Figs. A1a and A1b, $r = 1/\sqrt{2}$ and $r = \sqrt{2}/5$, respectively. For the larger spacing between peaks ($r = 1/\sqrt{2}$), passive performance is comparable to active for $dt = 0.002$. At smaller peak separation, however, passive diffusion does not resolve the peaks as well as active diffusion at the same value of the smallest examined $dt$.

### 2. Numerical Diffusion on Gaussian and Swiss Roll Toy Models

Here we examine the performance of passive, CLD, and active diffusion for a variety of 2D toy model distributions. For each distribution, we change the time step $dt$ of the reverse diffusion process and compare the diffusion generated samples to the target distribution. As mentioned before, denoising is turned off in the last step for these toy models (see Sec. A2 6).

Fig. A2 examines the same distributions that were tested with the analytic score model (Sec. 3 A and Sec. A3 1). Passive diffusion begins to resolve the positions of the larger features at $dt = 0.1$. CLD and active diffusion perform better than passive diffusion for $dt = 0.01$ and $dt = 0.002$.

Fig. A3 examines the Swiss roll distributions. For large $dt$ (= 0.1) all the methods, passive, active and CLD fail to resolve the coarse (locations of the swiss rolls) and finer details (the swiss roll spirals) of the distribution. At $dt = 0.01$, CLD and active diffusion both resolve the position of the Swiss rolls, and active diffusion begins to capture the spiral features. At $dt = 0.002$, CLD captures the spiral features of the Swiss rolls, while passive diffusion only shows faint traces of spiral structure (as evidenced in the point density indicated by green points in Fig. A3a).

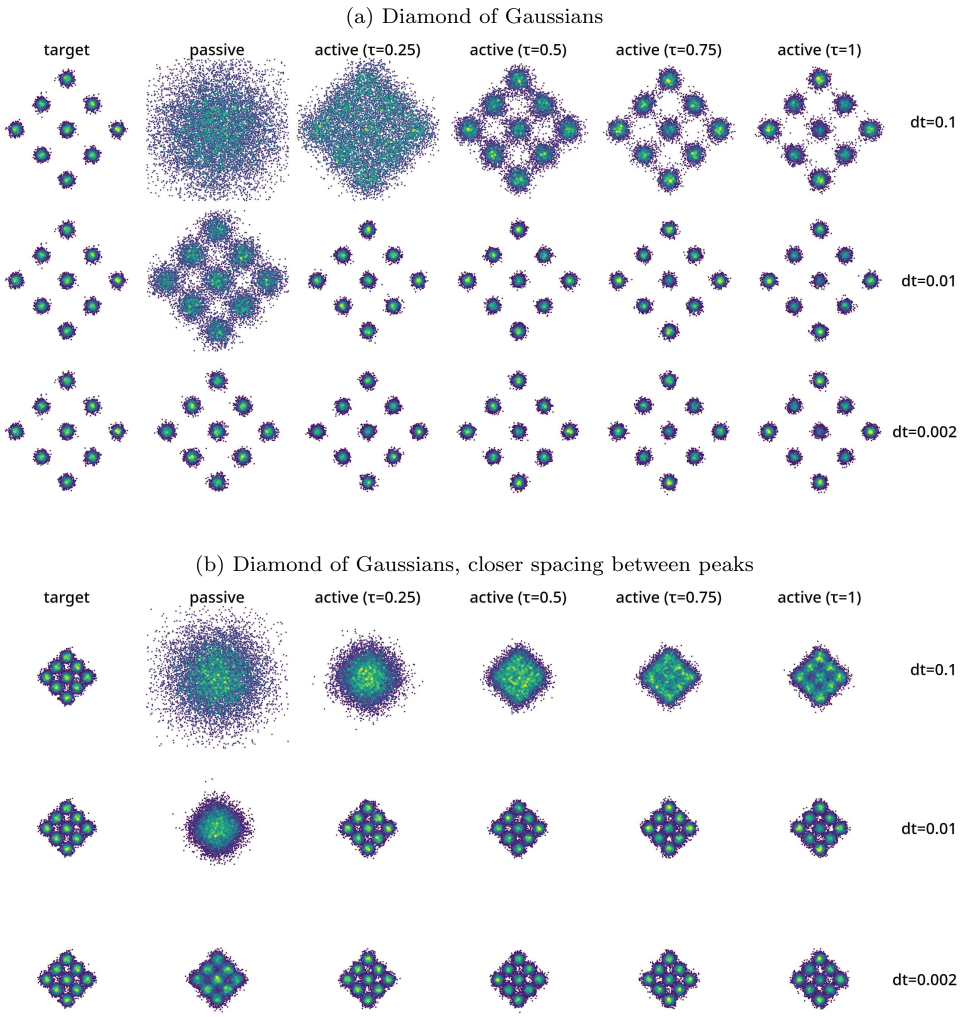


**FIG. A1:** Analytic score function for two sets of 9 Gaussian peaks with standard deviation $\sigma = 0.04$ but with different spacings between peaks. Gaussian peaks with spacing (a) $r = 1/\sqrt{2}$ and (b) $r = \sqrt{2}/5$. Increasing the correlation time $\tau$ improves the performance of active diffusion, which is most evident at the largest $dt$ examined here. At large $dt$, active diffusion outperforms passive diffusion. (a) At $dt = 0.002$, passive and active diffusion resolve the individual peaks to a similar degree. (b) Decreasing the spacing between peaks also decreases the resolution of peaks for passive diffusion for $dt = 0.002$, while active diffusion is not as affected by the change in distance between peaks.

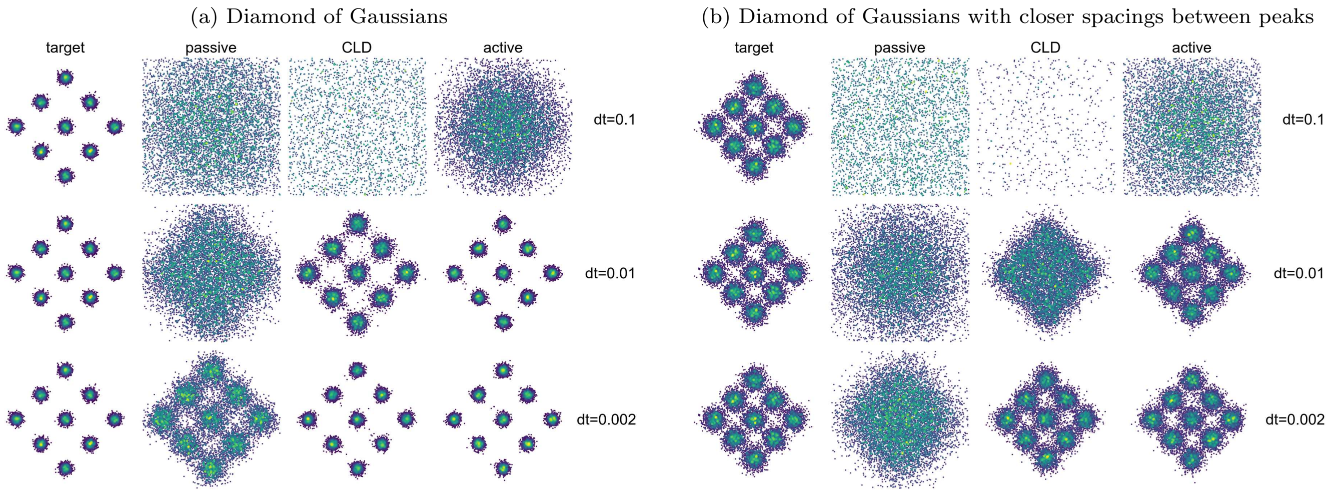


**FIG. A2:** Performance of various models with the score function approximated by a a neural network for Gaussian mixture target distributions at two peak separation distances ((a) $r = 1/\sqrt{2}$ and (b) $r = \sqrt{2}/5$).

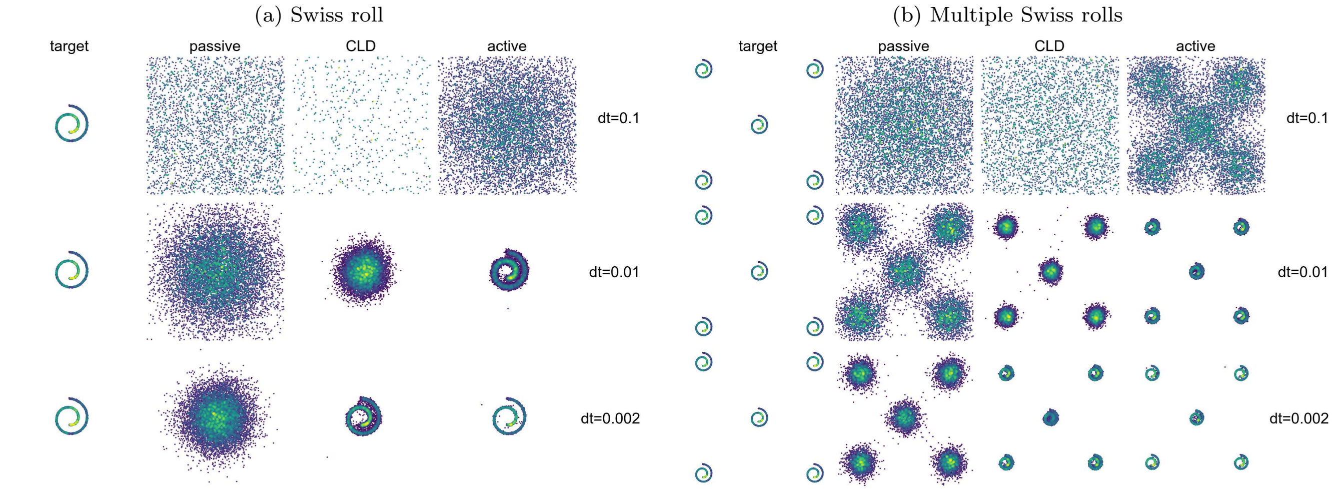


**FIG. A3:** Performance of various models with the score function approximated by a a neural network for distributions consisting of a single (a) and multiple (b) Swiss rolls.

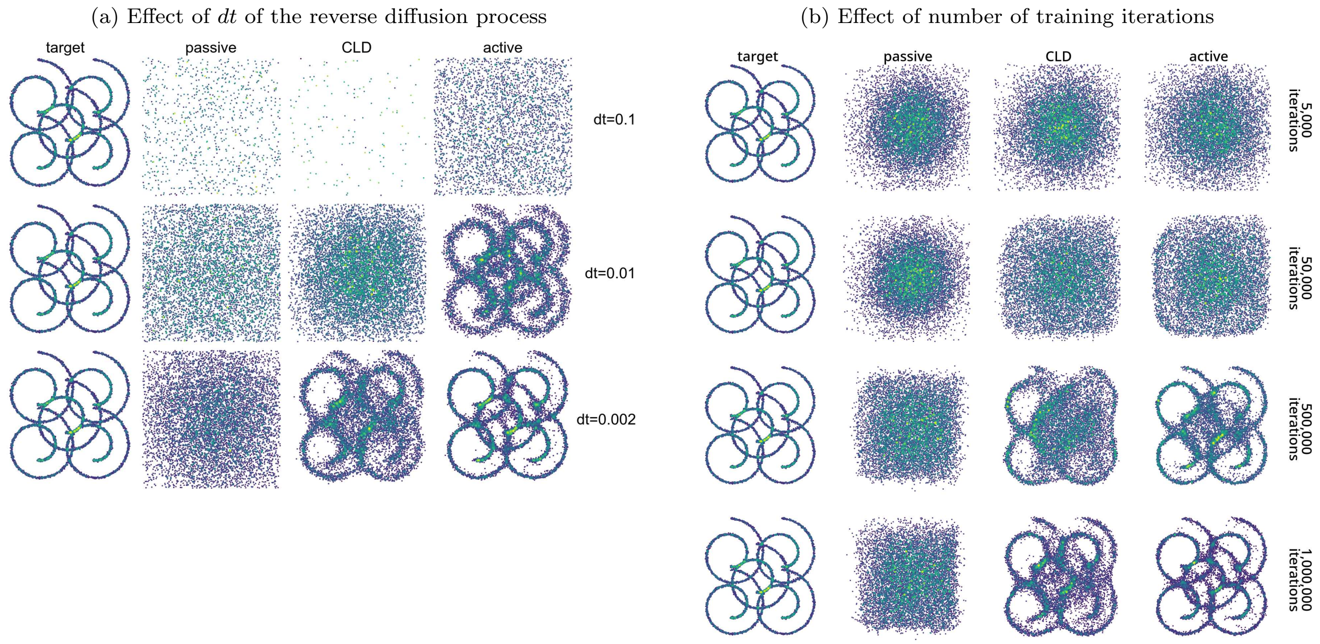


**FIG. A4:** Performance of various models with the score function approximated by a a neural network for distributions consisting of five overlapping Swiss rolls. (a) Effect of $dt$ on generated samples. (b) Effect of number of training iterations on generated samples.

## A4. ALANINE DIPEPTIDE TRAINING DATA GENERATION

Using GROMACS v2022.4 [38, 39], we prepared the alanine dipeptide system using the amber03 force field [40], with explicit solvation using TIP3P [41] water molecules in a cubic box with length 1.2 nanometers with periodic boundary conditions in all directions. We also added NaCl at 0.15 molar concentration to represent physiological conditions. All electrostatics were treated using the Particle-Mesh-Ewald (PME) method [42] in GROMACS. We energy minimized the system for 50,000 steps using the steepest descent algorithm. After energy minimization, we held the alanine dipeptide position fixed and equilibrated the system under constant number, volume, and temperature (NVT) ensemble for 10 nanoseconds. We used the modified Berendsen thermostat (velocity-rescale) to control the temperature. [43] We then equilibrated the system further under the constant number, pressure, and temperature (NPT) ensemble for another 10 nanoseconds. Here, we maintained the temperature using the modified Berendsen thermostat and maintained the pressure using the Parrinello-Rahman barostat. [44] Pressure was maintained isotropically in X, Y, and Z directions. For both NVT and NPT equilibration, we maintained a temperature of 300 K. For NPT equilibration, we maintained a pressure of 1 bar, using a 2 ps time constant for the Parrinello-Rahman barostat. In all cases, we used the leap-frog molecular dynamics integrator with a 2 fs timestep within GROMACS.

Using the resulting structure of the equilibrated system, we ran 1 microsecond of unbiased, brute force NPT dynamics on the alanine dipeptide, controlling temperature and pressure with the modified Berendsen thermostat and Parrinello-Rahman barostats respectively. To control the size of the dataset, we extract the conformation of the alanine dipeptide at every picosecond, leading to 1,000,000 conformations for 1 microsecond of simulation.

## A5. ALANINE DIPEPTIDE DIFFUSION

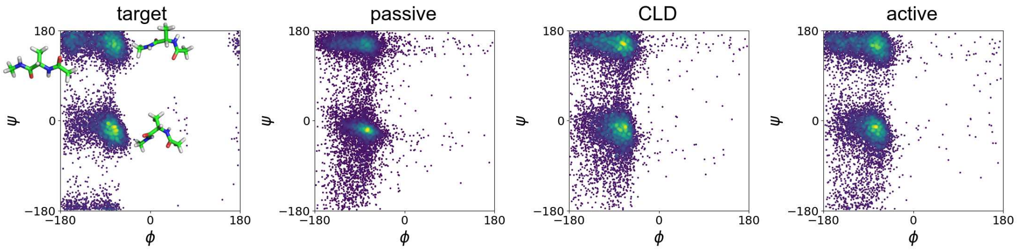


**FIG. A5:** Ramachandran plots ($\phi$, $\psi$) in degrees for 1 $\mu$s of sampling for a water-solvated alanine dipeptide (left) and corresponding diffusion generated samples with passive (center left), CLD (center right), and active ($\tau = 0.5$) (right).

The score model for the 2D distribution $(\phi, \psi)$ of alanine dipeptide was learned using a multi-layer perceptron with 4 hidden layers with 128 nodes each. The same architecture was used for the other 2D toy models. The 25D score model was learned using a U-net. The dataset was transformed into a $5 \times 5$ "image" to be used as input in the NN architecture used for image diffusion.

Although the alanine dipeptide molecule has 28 unique parameters (bond lengths, bond angles, and dihedral angles), we reduce the dataset dimensionality by averaging some bond lengths. This reduction to 25 dimensions allowed us to treat the data like an image and use existing code functionality to demonstrate that active diffusion also outperforms passive diffusion for higher-dimensional toy datasets. As with the 2D alanine dipeptide dataset (Fig. A5), active diffusion performs better than passive diffusion or CLD at a lower number of iterations.

## A6. SPECIATION WITH ACTIVE NOISE

From Eq. (A8) we integrate the $\boldsymbol{\eta}$ and $\boldsymbol{\eta}_0$ degrees, obtaining

$$P(\mathbf{x};t) = \int P(\mathbf{x}_0) P(\mathbf{x}|\mathbf{x}_0;t) \tag{A48}$$

$$= \int P(\mathbf{x}_0) \exp\left(-\frac{(\mathbf{x}-\mathbf{x}_0 e^{-t})^2}{2\Delta_t^a}\right) \tag{A49}$$

$$= \exp\left(-\frac{\mathbf{x}^2}{2\Delta_t^a} + g(\mathbf{x})\right) \tag{A50}$$

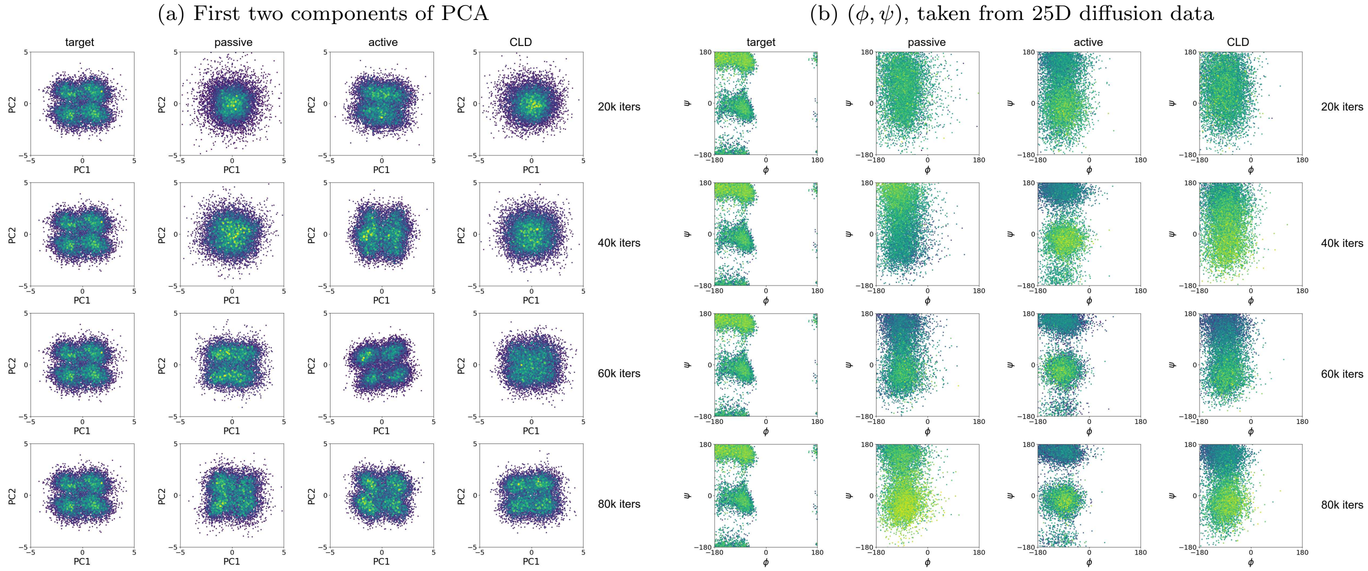


**FIG. A6:** Effect of increasing number of training iterations on performance of 25D diffusion. (a) The first two principal components of training data (left column) and diffusion-generated data. Active diffusion PCA plot begins to resemble the training data at a lower number of training iterations. (b) Plot of dihedral angles generated by 25D diffusion. Active plot begins to resemble training data faster than passive diffusion or CLD.

where $\mathbf{x}_0$ is the data at $t = 0$, and $g(\mathbf{x})$ and $\Delta_t^a$ are defined as

$$g(\mathbf{x}) = \log\left[\int D\mathbf{x}_0 P(\mathbf{x}_0)\exp\left(-\frac{\mathbf{x}_0^2 e^{-2t}}{2\Delta_t^a} - \frac{e^{-t}\mathbf{x}\cdot\mathbf{x}_0}{\Delta_t^a}\right)\right] \tag{A51}$$

$$\Delta_t^a = \frac{T_a}{1+\tau} + \frac{\tau}{1-\tau}\left[\frac{2\tau e^{-(1+\frac{1}{\tau})t}}{1+\tau} - e^{-2t}\right] \tag{A52}$$

In the limit of large time, one can expand $g(\mathbf{x})$ in terms of correlation functions. This yields

$$g(\mathbf{x}) = \frac{e^{-t}}{\Delta_t^a}\sum_{i=1}^{d} x_i \langle x_{0,i}\rangle + \frac{e^{-2t}}{2\Delta_t^a}\sum_{i,j=1}^{d} x_i x_j [\langle x_{0,i}x_{0,j}\rangle - \langle x_{0,i}\rangle\langle x_{0,j}\rangle] + \mathcal{O}\big((xe^{-t})^3\big) \tag{A53}$$

$$\text{where } \langle\cdot\rangle = \mathbb{E}_{P(\mathbf{x_0})\exp\left(-\frac{\mathbf{x_0}^2 e^{-2t}}{2\Delta_t^a}\right)}[\cdot] \tag{A54}$$

$$\log(P_t(\mathbf{x})) = C + \frac{e^{-t}}{\Delta_t^a}\sum_{i=1}^{d} x_i\langle x_{0,i}\rangle - \frac{1}{2\Delta_t^a}\sum_{i,j=1}^{d} x_i M_{ij} x_j + \mathcal{O}\big((\mathbf{x}e^{-t})^3\big) \tag{A55}$$

$$M_{ij} = \delta_{ij} - \frac{e^{-2t}}{\Delta_t^a}[\langle x_{0,i}x_{0,j}\rangle - \langle x_{0,i}\rangle\langle x_{0,j}\rangle] \tag{A56}$$

The speciation time is given by the time when the curvature of $\log(P_t)$ changes shape. The matrix $M$ provides a quadratic form which helps $\log(P_t)$ change shape. The time for speciation, $t_s^a$, is thus the time when the largest eigenvalue of $M$ crosses 0. At large times, the correlations in $M$, i.e., $\langle x_{0,i}x_{0,j}\rangle - \langle x_{0,i}\rangle\langle x_{0,j}\rangle$, can be substituted with

the true covariance matrix of the target distribution, $C_0$. This leads to,

$$e^{-2t_s^a} \cdot \max_{\lambda}(\mathrm{C}_0) = \Delta^a_{t\to\infty} \tag{A57}$$

$$t_s^a = \frac{1}{2}\log\left(\frac{\max_\lambda(\mathrm{C}_0)(1+\tau)}{T_a}\right) \tag{A58}$$

where the notation $\max_\lambda(\cdot)$ denotes the maximum eigenvalue of $(\cdot)$.

## A7. BELIEF PROPAGATION ON HIERARCHICAL DATA MODEL

In this section we provide details about the hierarchical model and belief propagation algorithm on the graph. We closely follow Ref. [23] and modify the analysis for our specific need. Natural images generally show a hierarchical structure with basic low level features like linea, patches, gradients etc being composed to form high level features which ultimately lead to the final image. To mimic this structure, a random hierarchical model (RHM) is developed.

### 1. Generation of Data

The RHM defines a tree-like generative model with the following parameters:

1. $L$ - Number of levels of the tree
2. $s$ - Branching factor i.e. the number of children of every parent node
3. $v$ - the alphabet size (each latent variable can take one of the $v$ values in $\{0, 1, ..., v-1\}$)
4. $m$ - the number of production rules per symbol

Level $L$ is the root node and level 0 are the leaf nodes which are the observations. The production rules determine how data is generated. Each parent symbol $b$ ($\in \{0, 1, .., v-1\}$) at level $l$ can produce a certain combination of children. We encode this as a set $T_b^l$ of allowed tuples defined as,

$$T_b^l = \{\mathbf{t}^{(1)}, \mathbf{t}^{(2)}, .., \mathbf{t}^{(m)}\}, \text{ m such tuples} \tag{A59}$$

$$\mathbf{t}^{(i)} = (t_1^{(i)}, t_2^{(i)}, .., t_s^{(i)}),\ t_k^{(i)} \in \{0, 1, ..., v-1\} \forall k,\ k = \{1, 2, .., s\} \tag{A60}$$

The alphabets are represented as one-hot encoding in the $\mathbb{R}^v$ space. The dimensions of the observation (data) which corresponds to the leaf layer is thus given as, $\mathbb{R}^{v\times s^L}$. Data is generated in the following way:

1. Sample the root node uniformly from the alphabets $\{0, 1, ..., v-1\}$
2. For every layer, sample uniformly the production rules corresponding to the parent symbol and generate the child tuple
3. iterate this till you reach the leaves
4. represent the data as one-hot encoding of the alphabet at every index where the index ranges from 1 to $s^L$

### 2. Optimal Denoising with Belief Propagation

We use Bayes optimal denoising for the RHM. The data ($\vec{x}$) is first taken through a noising process, $\vec{x}(0) \to \vec{x}(t)$, then the probability, $P(\vec{x}(0)|\vec{x}(t))$ is computed exactly. Using this we compute the marginal probabilities of all the latent variables at all the layers using the message passing algorithm. Then we check if the marginals correctly predict the true labels at every layer of the data generation process.

### 1. Belief Propagation

In the case of RHM, the leaf nodes correspond to the input variables when messages(beliefs) are passed upwards and the root node is considered as the input variable when the messages are passed down. The factor nodes correspond to the production rules used for the creation of the data. During the upward pass, every parent collects the messages from its children and updates its beliefs and sends a message upward. This is iteratively performed till one reaches the root. Then in the downward phase, starting from the root, every node sends a downward message to its children and they update their beliefs and send subsequent messages. This is iteratively performed till one reaches the leaves. The beliefs during the upwards and downward phase are stored. The marginal is given as the product of the upward and downward beliefs.

Define $\mathbf{X}^{(0)}$ as the data vector and $\mathbf{X}^{(l)}$ be the high-level variable corresponding to the data at layer $\ell$. Let $\psi^{(\ell)}$ be any factor node connecting an $s$-tuple of low-level variables at layer $\ell-1$, $\{\vec{X}_i^{(\ell-1)}\}_{i\in[s]}$, to a high-level variable $X_1^{(\ell)}$ at layer $\ell$. Without loss of generality, to lighten the notation, we rename the variables as $Y = X_1^{(\ell)}$, taking values $y \in \mathcal{A}$, and $X_i = X_i^{(\ell-1)}$, each taking values $x_i \in \mathcal{A}$. For each possible association $y \to x_1, \dots, x_s$, the factor node $\psi^{(\ell)}(y, x_1, \dots, x_s)$ takes values

$$\psi^{(\ell)}(y, x_1, \dots, x_s) = \begin{cases} 1, & \text{if } \{x_1, x_2..., x_s\} \in T_y^l \\ 0, & \text{otherwise.} \end{cases}$$

The BP upward and downward iterations for the (unnormalized) upward and downward messages respectively read

$$\begin{aligned} \tilde{v}_\uparrow^{(\ell+1)}(y) &= \sum_{x_1,\dots,x_s \in \mathcal{A}^{\otimes s}} \psi^{(\ell+1)}(y, x_1, \dots, x_s) \prod_{i=1}^{s} v_\uparrow^{(\ell)}(x_i), \\ \tilde{v}_\downarrow^{(\ell)}(x_1) &= \sum_{\substack{x_2,\dots,x_s \in \mathcal{A}^{\otimes(s-1)} \\ y\in\mathcal{A}}} \psi^{(\ell+1)}(y, x_1, \dots, x_s) \\ &\quad \times v_\downarrow^{(\ell+1)}(y) \prod_{i=2}^{s} v_\uparrow^{(\ell)}(x_i), \end{aligned} \tag{5}$$

where $v_\rho^{(\ell)}(x) = \frac{\tilde{v}_\rho^{(\ell)}(x)}{\sum_{x'} \tilde{v}_\rho^{(\ell)}(x')}, \rho \in \{\uparrow, \downarrow\}$. The downward iteration, reported for $x_1$, can be trivially extended to the other variables $x_i$ by permuting the position indices. The values of $v_\uparrow^{(0)}(x_i)$ and $v_\downarrow^{(L)}(y)$ are set by the initial conditions.

### 2. Initialization of the leaf and root nodes

For the root nodes, we initialize the downward messages, $v_\downarrow^{(L)}(y) = 1/v$, which corresponds to a uniform prior over the possible classes $\{0, 1, ..., v-1\}$.

For the leaf nodes, the initialization of the upward messages is a little involved. As defined previously, data is $\mathbf{X}^{(0)}$. The data is in fact a matrix of $s^L$ columns of vectors $\vec{X}_i^{(0)} \in \mathbb{R}^v$. Thus we can write without loss of generality $X_i^{(0)} = e_\gamma$, with $e_\gamma$ a canonical basis vector with 1 in position $\gamma$ and 0 everywhere else. Its continuous diffusion process takes place in $\mathbb{R}^v$: Given the value $X_i^{(0)} = x_i(t)$, we can compute the probability of its starting value $p(x_i(0)|x_i(t))$ using Bayes formula. This computation is performed independently for each input variable $i$, and therefore does not take into account the spatial correlations given by the generative model. The probabilities of Eq. 4 are used to initialize the BP upward messages $v_\uparrow^{(0)}(x_i) = p(x_i(0)|x_i(t))$ at the input variables. In our active case, we have an additional $\eta^{(0)}$ associated with the data which leads to the computation of $p(x_i(0)|x_i(t), \eta_i(t))$ instead of $p(x_i(0)|x_i(t))$. From the passive and active processes defined in Eq. 1, 5, 6, A8, one can easily compute these conditional probabilities

after some messy algebra,

$$\text{Passive: } p(x(0) = e_\mu|x(t)) = \frac{1}{Z}\exp\left(\frac{e^{-t}}{T(1-e^{-2t})}x_\mu(t)\right) \tag{A61}$$

$$Z = \sum_\lambda p(x(0) = e_\lambda|x(t)) \tag{A62}$$

$$\text{Active: } p(x(0) = e_\mu|x(t),\eta(t)) = \frac{1}{Z}\exp\left(-\frac{(m_{11}\alpha - m_{12}e^{-t/\tau})e^{-kt}[(m_{11}\alpha - m_{12}e^{-t/\tau})x_\mu(t) + (m_{22}e^{-t/\tau} - m_{12}\alpha)\eta_\mu(t)]}{\Delta[(\tau/T_a)\Delta + m_{11}\alpha^2 - 2m_{12}e^{-t/\tau}\alpha + m_{22}e^{-2t/\tau}]}\right. \tag{A63}$$

$$\left. + \frac{e^{-kt}(m_{11}x_\mu(t) - m_{12}\eta_\mu(t))}{\Delta}\right) \tag{A64}$$

where $m_{11}, m_{12}, m_{22}$ are as defined in Eq. A11,

We run three different simulations, one with passive noising process, one with active noising process and a third set with a passive temperature "equivalent" to the active temperature. The equivalence is set by the variance of the data dimension at the end of the nosiing process. For passive noise it is given by the passive temperature, $T$. For the active nosiing process, the final variance on the data dimension is given by, $T_a/(k(1+k\tau))$. The third set of simulation is carried out using a passive noising process with $T = T_a/(k(1+k\tau))$.

## 8. CIFAR-10 DIFFUSION

We use a DDPM/NCSN++-style U-Net backbone on 32×32 CIFAR-10 images. The base width is 128 with channel multipliers $(1, 2, 2, 2)$, yielding feature dims $\{128, 256, 256, 256\}$ across four resolution levels, each with 4 residual blocks. Timesteps are embedded with sinusoidal features (dim 256) followed by an MLP (4× expansion) and injected into each residual block. Each block uses GroupNorm, SiLU, and two 3×3 convolutions (with a 1×1 skip when needed). Downsampling uses a 3×3 stride-2 conv; upsampling uses nearest-neighbor interpolation followed by a 3×3 conv, with skip concatenation. Self-attention is applied at 16×16 resolution, and the bottleneck is ResBlock–Attn–ResBlock. The output head is GroupNorm+SiLU+3×3 conv. The passive model uses 3 input/output channels (RGB). The active model uses 6 input/output channels (RGB concatenated with 3 $\eta$ channels).
